# Anti-SARS-CoV-2 Small Molecule Targeting of Oxysterol-Binding Protein (OSBP) Activates Cellular Antiviral Innate Immunity

**DOI:** 10.1021/acsinfecdis.4c00631

**Published:** 2025-04-21

**Authors:** Bharathiraja Subramaniyan, Emily C. Falcon, Andrew R. Moore, Jason L. Larabee, Susan L. Nimmo, Jorge L. Berrios-Rivera, William J. Reddig, Earl L. Blewett, James F. Papin, Matthew S. Walters, Anthony W. G. Burgett

**Affiliations:** † Department of Medicine, Section of Pulmonary, Critical Care & Sleep Medicine, University of Oklahoma Health Sciences Center, Oklahoma City, Oklahoma 73104, United States; ‡ Department of Pharmaceutical Sciences, University of Oklahoma Health Sciences Center, Oklahoma City, Oklahoma 73117, United States; § Department of Microbiology and Immunology, University of Oklahoma Health Sciences Center, Oklahoma City, Oklahoma 73104, United States; ∥ Department of Biochemistry and Microbiology, Oklahoma State University Center for Health Sciences, Tulsa, Oklahoma 74107, United States; ⊥ Department of Pathology, Division of Comparative Medicine, University of Oklahoma Health Sciences Center, Oklahoma 73104, United States; # Stephenson Cancer Center, University of Oklahoma Health Sciences Center, Oklahoma City, Oklahoma 73104, United States

**Keywords:** oxysterol-binding protein (OSBP), OSW-1, antiviral compounds, SARS-CoV-2, rhinovirus, innate antiviral immune response

## Abstract

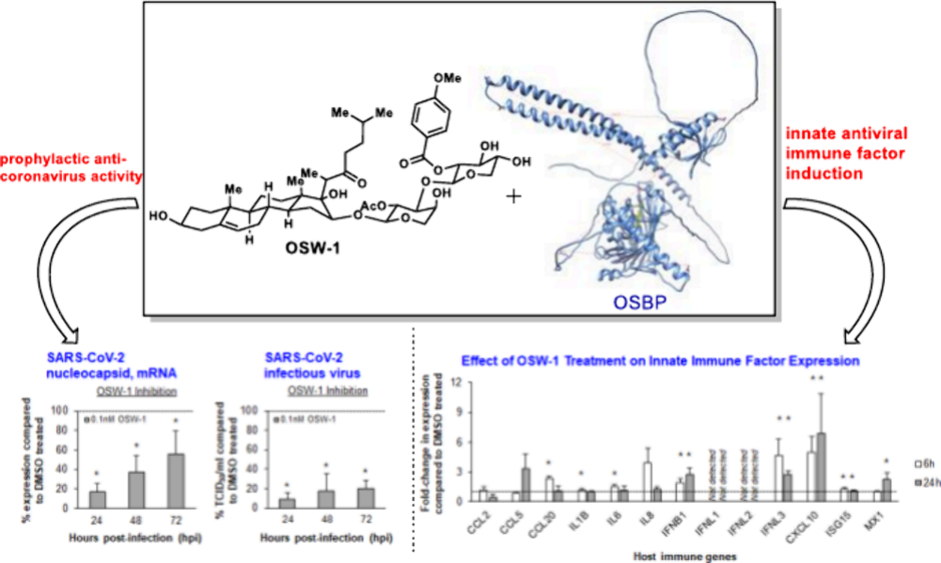

Human oxysterol-binding protein (OSBP) is a potentially druggable mediator in the replication of a broad spectrum of positive-sense (+) single-stranded RNA (ssRNA) viruses, including members of the *Picornaviridae*, *Flaviviridae*, and *Coronaviridae*. OSBP is a cytoplasmic lipid transporting protein capable of moving cholesterol and phosphoinositides between the endoplasmic reticulum (ER) and Golgi, and the ER and lysosome. Several structurally diverse antiviral compounds have been reported to function through targeting OSBP, including the natural product compound OSW-1. Our prior work shows that transient OSW-1 treatment induces a reduction in OSBP protein levels over multiple successive cell generations (i.e., multigenerational), with no apparent cellular toxicity, and the OSW-1-induced reduction of OSBP has antiviral activity against multiple (+)ssRNA viruses. This study extends these findings and establishes that OSW-1 has in vitro antiviral activity against multiple pathogenic (+)ssRNA viruses, including human rhinovirus (HRV1B), the feline coronavirus peritonitis virus (FIPV), human coronavirus 229E (HCoV-229E), and severe acute respiratory syndrome coronavirus 2 (SARS-CoV-2). We also demonstrate that OSW-1 treatment in human airway epithelial cells alters the expression of multiple antiviral innate immune mediators, including the interferon (IFN) related genes IFNB1, IFNL3, CXCL10, ISG15, and MX1. Furthermore, OSW-1 enhances the induction of specific components of type I and III IFN antiviral responses triggered by the RNA viral mimetic polyinosinic-polycytidylic acid (Poly IC). In summary, this study further demonstrates the importance of OSBP in (+)ssRNA virus replication and presents OSBP as a potential regulator of cellular antiviral innate immune responses.

The recent COVID-19 pandemic caused by severe acute respiratory syndrome coronavirus 2 (SARS-CoV-2) underscores the urgent need to develop new modalities for the therapeutic treatment of pathogenic RNA viruses.^1^ Effective antiviral drugs with broad-spectrum activity against many different viral pathogens could provide an immediate mode of therapeutic intervention in emerging RNA virus pandemics.^1^

One potential target for host-directed antiviral drug targeting is the nonenzymatic lipid-binding protein oxysterol-binding protein (OSBP).^2−7^ OSBP is capable of transporting lipids between organelles at membrane contact sites (MCS).^8^ While the cellular function of OSBP, and the closely related OSBP-related proteins (ORPs), are not fully defined, OSBP is reported to coordinate the transfer of phosphoinositide-4-phosphate (PI4P) and cholesterol between the endoplasmic reticulum (ER) and Golgi,^9^ and transfer cholesterol and PI4P between the ER and the lysosome membranes to activate mTORC1.^10,11^ Recent studies have demonstrated that OSBP is an important host factor required for the replication of a broad range of positive-sense (+) single-stranded RNA (ssRNA) viruses.^6,12−15^ This includes viruses of the nonenveloped *Picornaviridae* family (e.g., multiple viruses in the *Enterovirus* genus, human rhinovirus, and Encephalomyocarditis virus)^6,12,13^ and enveloped *Flaviviridae* (e.g., Zika virus, Dengue virus and Hepatitis C virus).^14,15^ A role of OSBP in *Coronaviridae* viral replication has not been conclusively shown, although feline coronavirus is reported to be inhibited by a putative OSBP-targeting antiviral compound.^16^ As a lipid transporter, OSBP is reported to help form the membrane-wrapped viral replication organelles (VRO), which are essential components of the infective life cycle of many (+)ssRNA viruses.^17^ VROs are cellular structures used to store viral components and assemble virion particles, safe from the surveillance and antiviral defenses of cellular innate immune factors.^17^ A vital role of OSBP in the formation of VROs is the postulated antiviral mechanism of action of OSBP-targeting compounds.^17^ In multiple cell lines tested, a reduction in OSBP protein levels has minimal impact on cell proliferation or viability.^2−4,6^

Based on its role in regulating replication of multiple (+)ssRNA viruses, OSBP may be a viable cellular target for developing broad-spectrum antivirals against many RNA viral pathogens. Several structurally diverse antiviral compounds have been reported to function through targeting OSBP, namely, OSW-1,^2,3^ T-00127-HEV2 (aka THEV),^4,18^ TTP-8307 (TTP),^7^ and itraconazole (ITZ),^6,15^ with each compound having differing effects on OSBP.^2^ OSW-1 is a natural product isolated from the bulbs of *Ornithogalum saundersiae* that targets OSBP and its closely related paralog ORP4 (also known as OSBP2).^19,20^ OSW-1 binds OSBP and ORP4 with low nanomolar affinity; with a *K*_i_ = 16 ± 4 nM for OSBP and a *K*_i_ = 71 ± 6 nM for ORP4.^21^ OSBP and ORP4 have high shared homology and both bind sterols and phosphoinositides, but despite these similarities, OSBP and ORP4 appear to have distinct cellular functions.^11^ OSBP, but not ORP4, transports lipid between organelles^9^ and indirectly regulates mTORC1 activity through lysosome lipid composition.^10^ The ORP4 cellular function is currently more enigmatic and less defined than OSBP.^11^ ORP4, but not OSBP, has been shown to promote proliferation in cancer cells.^22,23^ Cellular reduction of ORP4 is cytotoxic to rapidly dividing cancer cell lines.^22^ Based on the importance of ORP4 in aberrant cell proliferation, the anticancer activity of the OSW-1 compound is likely due to the targeting of ORP4 in cancer cells, not OSBP.^22,23^ ORP4 is reported to drive cancer cell proliferation through serving as a scaffold for PLCβ3.^23^ One report suggested ORP4 could also be involved in viral replication, but the cytotoxic effect of using RNAi to reduce ORP4 expression in transformed cells prevented a definite conclusion.^6^

Our previous works discovered that short-term, transient treatment of cells with low nanomolar concentrations of OSW-1 compound triggers a long-term reduction in OSBP protein levels, which can last multiple days and cellular generations after the end of compound exposure.^2,3^ ORP4 levels were not affected by the short-term transient OSW-1 treatment.^3^ Furthermore, the OSW-1-induced reduction of OSBP levels inhibits the replication of two pathogenic *Enteroviruses*.^2,3^ Building off this prior work, our results demonstrate that the OSW-1 compound, through targeting OSBP, inhibits the replication of multiple pathogenic (+)ssRNA viruses that cause respiratory disease in humans (human rhinovirus 1B (HRV1B), human coronavirus 229E (HCoV-229E) and SARS-CoV-2) and animals (feline infectious peritonitis virus (FIPV)). In addition, we demonstrate that the OSW-1-induced chemical knockdown of OSBP protein levels activates components of the antiviral innate immune response in airway epithelial cells, and the OSW-1 induced reduction of OSBP enhances the expression of specific components of the type I and III interferon (IFN) response following stimulation with the viral mimetic polyinosinic-polycytidylic acid (Poly IC). Our results present OSBP as a potential regulator of cellular antiviral innate immunity.

## Results

### OSW-1 Treatment Demonstrates Antiviral Activity against Human Rhinovirus and Pathogenic Coronaviruses

**Figure 1:**
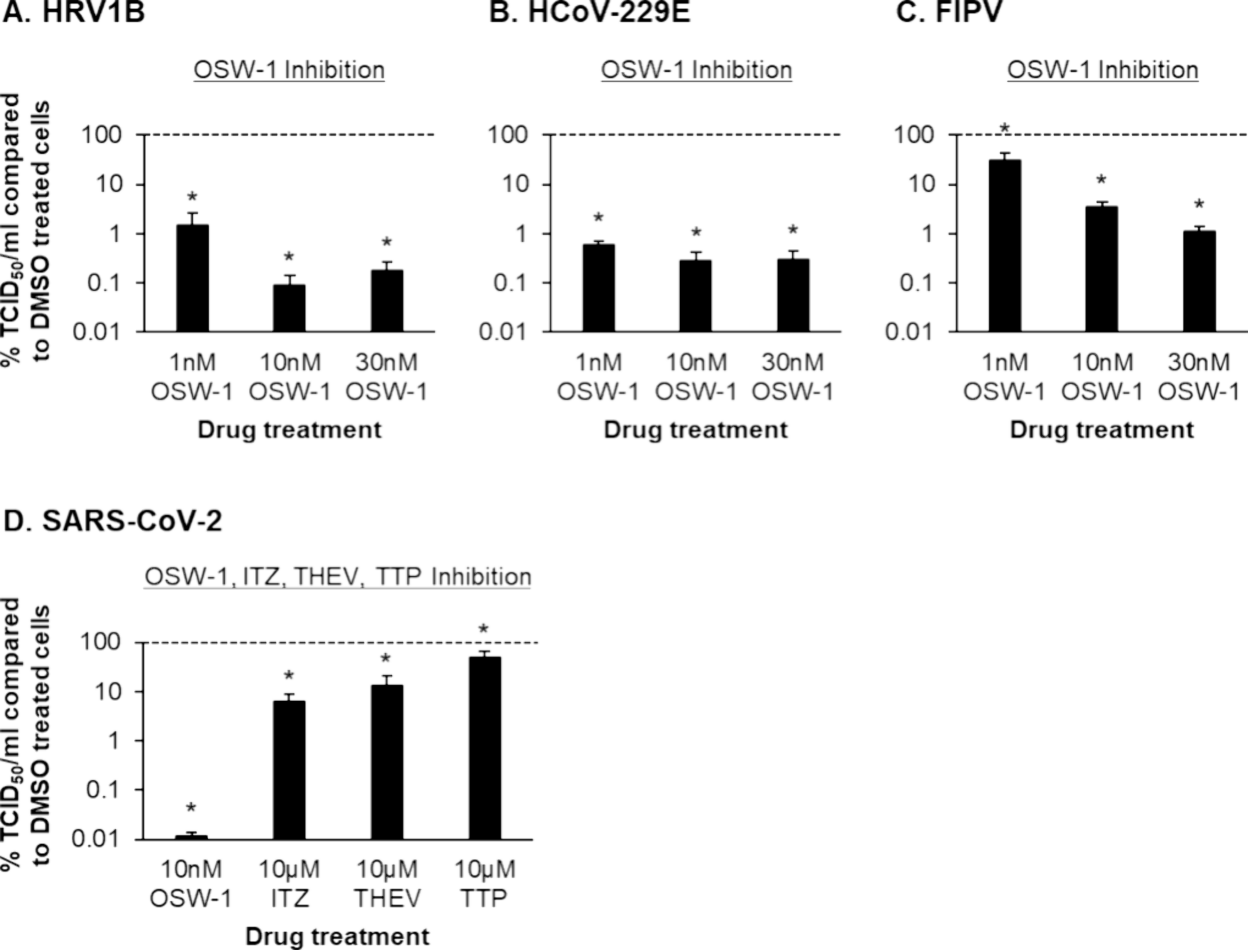
OSW-1 treatment inhibits replication of HRV1B, HCoV-229E, FIPV and SARS-CoV-2. (A) HRV1B-infected H1-HeLa cells treated with DMSO or OSW-1 from *n* = 3 independent experiments. (B) HCoV-229E infected MRC-5 cells treated with DMSO or OSW-1 from *n* = 4 independent experiments. (C) FIPV-infected CRFK cells treated with DMSO or OSW-1 from *n* = 3 independent experiments. (D) SARS-CoV-2-infected Vero E6 cells treated with DMSO, OSW-1, ITZ, THEV or TTP from *n* = 3 independent experiments. For A–D, the data are presented as percentage inhibition of virus replication by OSW-1, ITZ, THEV or TTP. For A–D, the bars represent the mean percentage of TCID50/mL in OSW-1, ITZ, THEV or TTP treated compared to DMSO treated cells, and the error bars indicate the standard error of the mean (SEM). * *p* ≤ 0.05.

Following on from our previous studies demonstrating the antiviral activity of OSW-1 against *Enteroviruses*,^2,3^ we tested the ability of OSW-1 treatment to suppress replication of a human rhinovirus (HRV1B) and human coronavirus (HCoV-229E), which are (+)ssRNA viruses that cause respiratory disease in humans.^24,25^ Using an OSW-1 treatment strategy shown to reduce OSBP protein levels and nontoxic doses,^2,3^ our results demonstrate that compared to the DMSO-vehicle control treated cells, OSW-1 treatment significantly (*p* ≤ 0.05) suppressed HRV1B replication at 1 nM (98.5%), 10 nM (99.9%) and 30 nM (99.8%) (Figure 1A). Similar results were also observed for HCoV-229E, with 1 nM (99.4%), 10 nM (99.7%), and 30 nM (99.7%) OSW-1 treatment significantly (*p* ≤ 0.05) suppressing virus replication (Figure 1B). OSW-1 treatment also significantly (*p* ≤ 0.05) suppresses the replication of the feline coronavirus peritonitis virus (FIPV), which is a feline coronavirus type II virus, in a dose-dependent manner at 1 nM (68.8%), 10 nM (96.5%), and 30 nM (98.9%) (Figure 1C). Finally, 10 nM OSW-1 treatment also significantly (*p* ≤ 0.05) suppresses SARS-CoV-2 replication (>99%) in VeroE6 cells (Figure 1D). Our previous study showed that other reported OSBP-targeting antiviral compounds, itraconazole (ITZ), T-00127-HEV2 (THEV), and TTP-8307 (TTP), inhibited enterovirus proliferation in cells with potency approximately 1000× less than OSW-1.^3^ Similarly, the OSBP-targeting ITZ, THEV, and TTP antiviral compounds dosed at 10 μM demonstrated less potent anti-SARS-CoV-2 activity compared to nanomolar levels of OSW-1 (Figure 1D). In summary, our data demonstrate that OSW-1 treatment has antiviral activity against multiple human and animal (+)ssRNA viruses that cause respiratory disease, including coronaviruses.

### Treatment of Immortalized Airway Epithelial Cells with OSW-1 Leads to the Knockdown of OSBP Protein Levels

**Figure 2:**
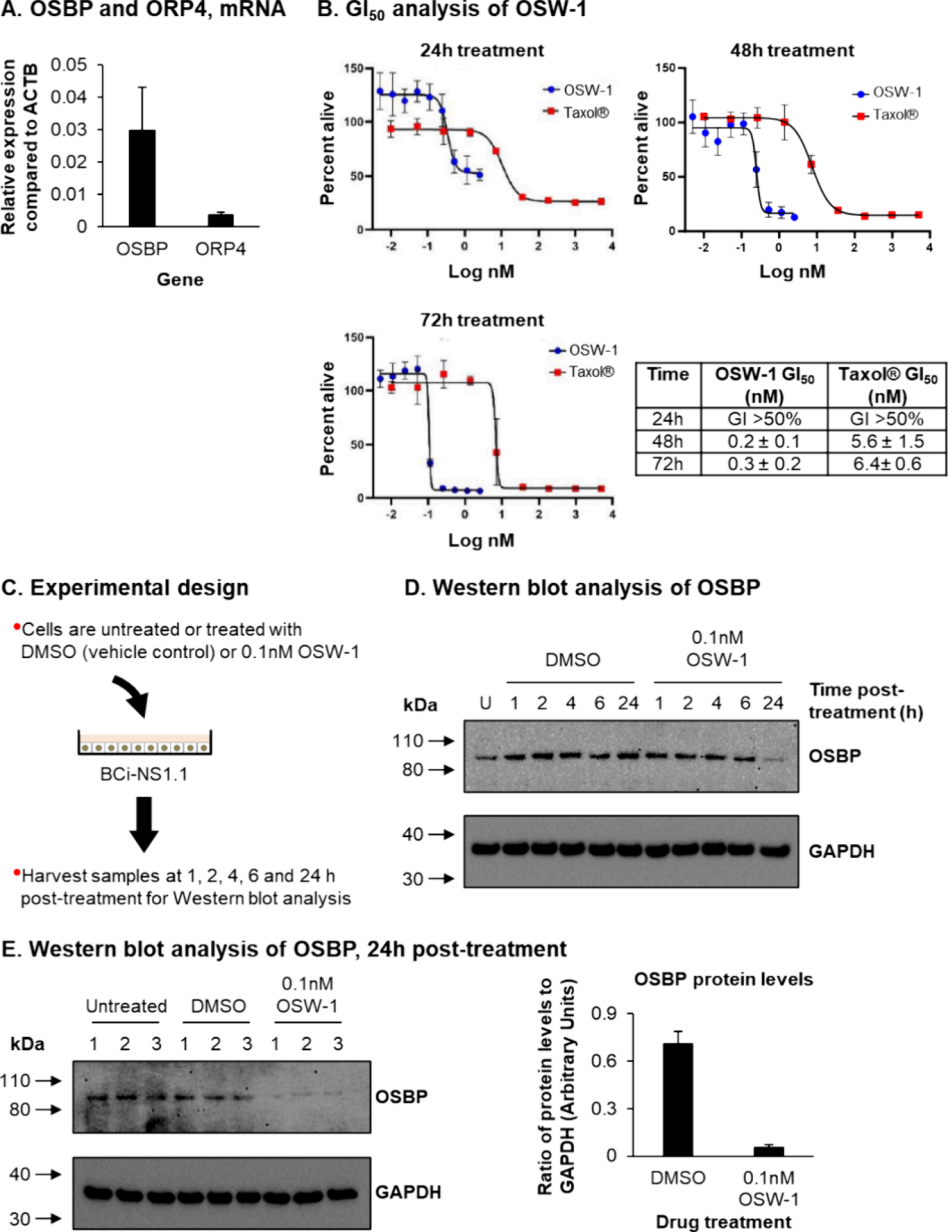
Treatment of BCi-NS1.1 cells with OSW-1 leads to a knockdown of OSBP protein levels. (A) qPCR of OSBP and ORP4 gene expression in BCi-NS1.1 cells. Bars represent mean expression in *n* = 3 replicates from a single experiment. Error bars indicate the SEM (B) CellTiter-Blue assay to determine the GI50 of OSW-1 and Taxol in BCi-NS1.1 cells at 24–72 h (h) post-treatment. Graphs are representative of one experiment with the mean value from *n* = 3 replicates shown for each concentration. Error bars indicate the standard deviation (SD). The table displays the mean and SD calculated from *n* ≥ 3 experiments for each compound and time point. (C) Experimental design for treatment of BCi-NS1.1 cells with OSW-1. (D) Western blot analysis of OSBP and GAPDH in whole cell lysates of BCi-NS1.1 cells either untreated or treated with DMSO or 0.1 nM OSW-1 for 1, 2, 4, 6, and 24 h. (E) Western blot analysis of OSBP and GAPDH in whole cell lysates of BCi-NS1.1 cells either untreated or treated with DMSO or 0.1 nM OSW-1 for 24 h. OSBP protein levels are shown with a representative Western blot shown as well as quantified values from three independent experiments, normalized to GAPDH protein levels for each treatment group. Bar represents mean levels from *n* = 3 replicates and error bars the SEM.

As the primary site of respiratory virus infection, airway epithelial cells are a more physiologically relevant model than many cell lines for studying the role of OSBP in regulating the replication of (+)ssRNA respiratory viruses in vitro*.*^26,27^ BCi-NS1.1 cells are an immortalized human airway epithelial cell line generated via hTERT expression in primary airway epithelial cells^28^ and have been used extensively to study the biology of multiple respiratory viruses, including rhinovirus, respiratory syncytial virus (RSV), influenza, and SARS-CoV-2.^29−31^ Importantly, BCi-NS1.1 cells express both the OSBP and ORP4. (Figure 2A). CellTiter-Blue growth inhibition assays in the BCi-NS1.1 cells at 24, 48, and 72 h established 0.1 nM OSW-1 as a nontoxic dose that did not inhibit cellular growth or alter cellular morphology. As a positive control for cytotoxicity, BCi-NS1.1 cells were treated with the standard-of-care anticancer drug paclitaxel (Taxol) (Figure 2B).

To investigate the ability of OSW-1 treatment to reduce OSBP in BCi-NS1.1 cells, cells were either untreated, treated with DMSO vehicle, or treated with 0.1 nM OSW-1 for 1, 2, 4, 6, and 24 h before harvest for Western blot analysis (Figure 2C). The results demonstrate that 24 h of 0.1 nM OSW-1 treatment reduces OSBP levels by 92% compared to DMSO-treated cells (Figure 2D,E). The shorter durations of OSW-1 treatment (i.e., 1–6 h) had minimal effects on OSBP levels (Figure 2D). Importantly, 24 h of 0.1 nM OSW-1 treatment had no effect on the levels of ORP4 protein (see Supplementary Figure S-2), confirming the specific targeting of OSBP with our treatment strategy. In summary, these data identify a nontoxic OSW-1 dosing regimen that leads to an OSW-1-induced chemical knockdown of OSBP protein levels in the BCi-NS1.1 airway epithelial cells.

### OSW-1 Pretreatment Inhibits the Replication of SARS-CoV-2 in an Airway Epithelial Cell Line

**Figure 3:**
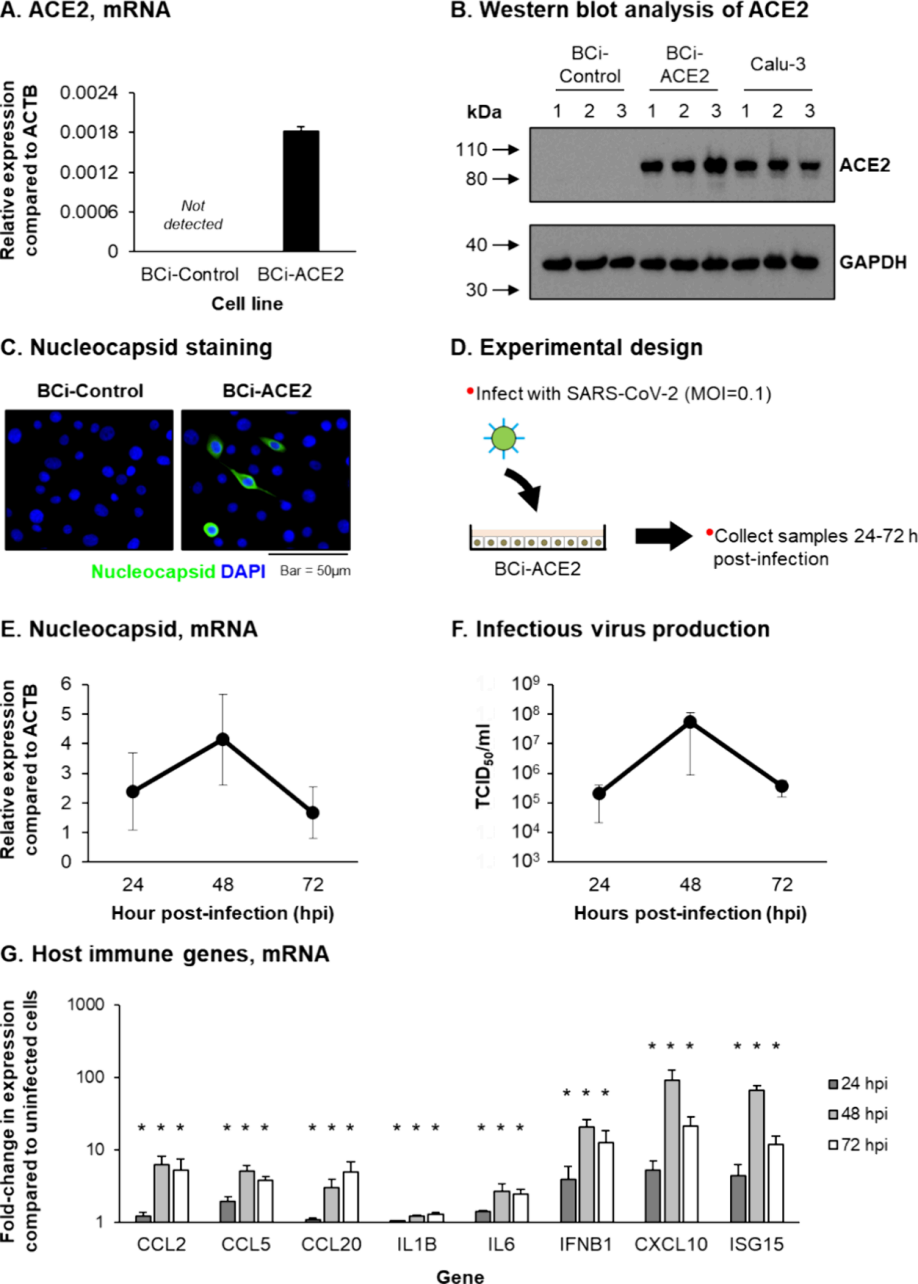
ACE2 expressing BCi-NS1.1 cells supports infection and replication of SARS-CoV-2. (A) qPCR of ACE2 gene expression in BCi-Control and BCi-ACE2 cells. Bars represent mean expression in *n* = 3 replicates from a single experiment. Error bars indicate the SEM (B) Western blot analysis of ACE2 and GAPDH in whole cell lysates of BCi-Control, BCi-ACE2 and Calu-3 cells. Data shown from a single experiment with *n* = 3 replicates per cell line. (C) Co-immunofluorescent staining of SARS-CoV-2 nucleocapsid (green) and cell nuclei (blue, DAPI) in BCi-Control and BCi-ACE2 cells infected with SARS-CoV-2. (D) Experimental design for infection of BCi-ACE2 cells with SARS-CoV-2. (E) qPCR of SARS-CoV-2 nucleocapsid gene expression at 24, 48, and 72 h post infection (hpi) in cultures of SARS-CoV-2 infected BCi-ACE2 cells. The circles represent the mean expression from *n* = 3 independent experiments and the error bars indicate the SEM (F) Infectious virus titer (TCID50/mL) at 24, 48, and 72 hpi in cultures of SARS-CoV-2 infected BCi-ACE2 cells. The circles represent the mean TCID50/mL from *n* = 3 independent experiments and the error bars indicate the SEM (G) qPCR of host immune gene expression (CCL2, CCL5, CCL20, IL1B, IL6, IFNB1, CXCL10 and ISG15) at 24 (dark gray bars), 48 (light gray bars) and 72 (white bars) hpi in cultures of SARS-CoV-2 infected BCi-ACE2 cells. Bars represent mean fold-change expression compared to uninfected cells in *n* = 3 independent experiments and error bars indicate the SEM * *p* ≤ 0.05.

To investigate the antiviral activity of OSW-1 against SARS-CoV-2 in a physiologically relevant cell model, we generated a stable BCi-NS1.1 cell line expressing the ACE2 receptor (BCi-ACE2) following infection with a replication-deficient lentivirus expressing ACE2 and subsequent neomycin selection of infected cells.^32^ In addition, a control cell line (BCi-Control) was created in tandem via infection with the empty vector control lentivirus that lacks the expression of human ACE2. Quantitative polymerase chain reaction (qPCR) analysis confirmed expression of ACE2 in the BCi-ACE2 cells, with no expression detected in the BCi-Control cells (Figure 3A). These results were validated at the protein level by Western blot analysis, with lysates from ACE2-expressing Calu-3 cells used as a positive control (Figure 3B). Infection of both BCi-Control and BCi-ACE2 cells with SARS-CoV-2 demonstrated functionality of the lentivirus-expressed ACE2 receptor, with only the BCi-ACE2 cells permissive of infection and staining positive for virus nucleocapsid (Figure 3C). The temporal kinetics of virus replication was further characterized in the BCi-ACE2 cells via quantification of virus nucleocapsid expression and production of infectious virus (Figure 3D). The results demonstrate that expression of nucleocapsid and production of infectious virus were readily detected at 24 h postinfection (hpi) and peaked at 48 hpi, before declining at 72 hpi (Figure 3E,F). qPCR analysis of the host innate immune response demonstrated significant (*p* ≤ 0.05) induction of multiple proinflammatory cytokines and chemokines (CCL2, CCL5, CCL20, IL1B, and IL6) and IFN-related genes (IFNB1, CXCL10, and ISG15) that correlate with the kinetics of virus replication (Figure 3G). Combined, these data support the use of BCi-ACE2 cells as a model to study SARS-CoV-2 replication.

**Figure 4:**
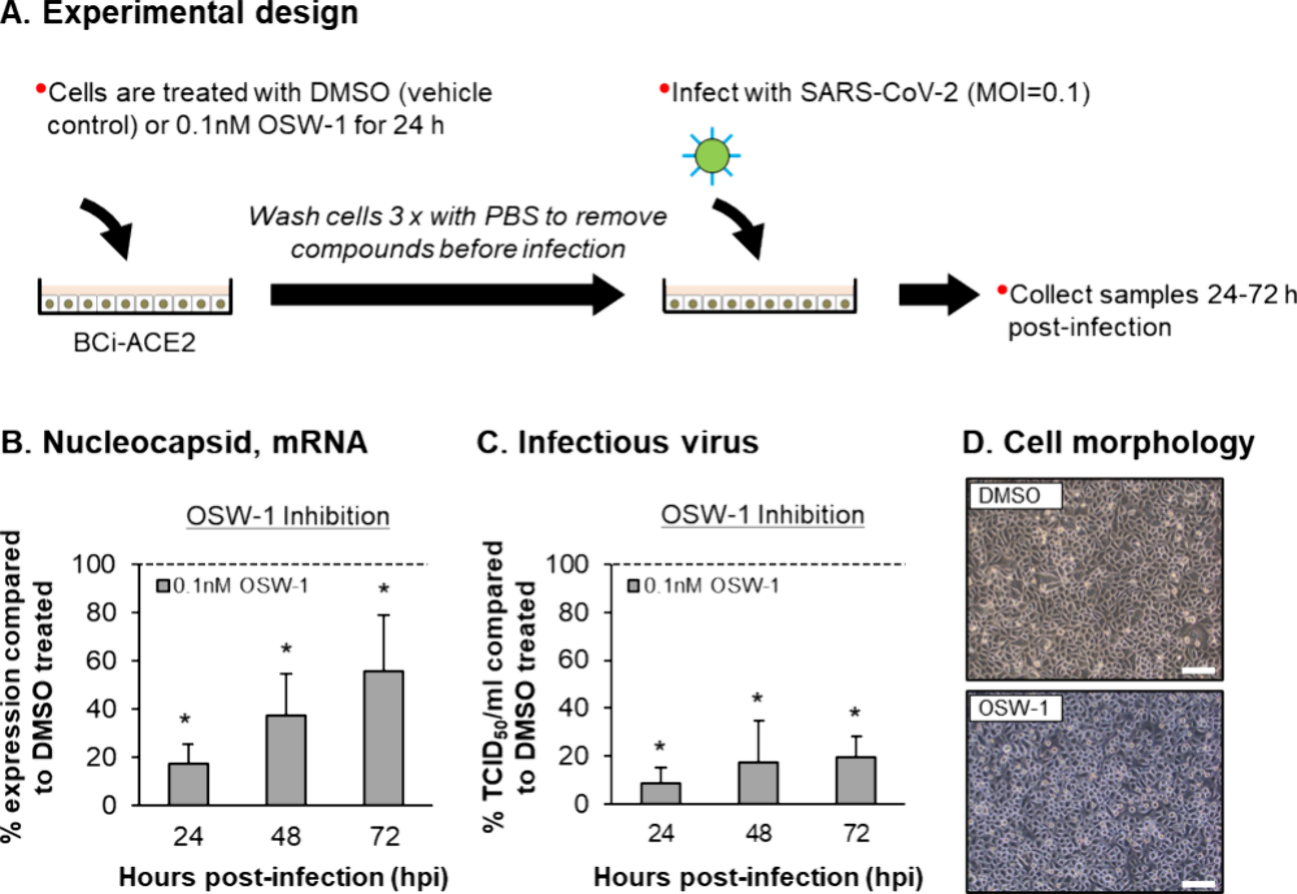
OSW-1 treatment inhibits the replication of SARS-CoV-2. (A) Experimental design for SARS-CoV-2 infection of BCi-ACE2 cells pretreated with DMSO or 0.1 nM OSW-1. (B) qPCR of SARS-CoV-2 nucleocapsid gene expression at 24, 48, and 72 h post infection (hpi) in cultures of SARS-CoV-2 infected BCi-ACE2 cells either pretreated with DMSO or 0.1 nM OSW-1 from *n* = 3 independent experiments. The data are presented as percentage inhibition of virus replication by OSW-1 The bars represent the mean percentage of expression in OSW-1 treated compared to DMSO treated cells and the error bars indicate the SEM (C) Infectious virus titer (TCID50/mL) at 24, 48, and 72 hpi in cultures of SARS-CoV-2 infected BCi-ACE2 cells either pretreated with DMSO or 0.1 nM OSW-1 from *n* = 3 independent experiments. The data are presented as percentage inhibition of virus replication by OSW-1. The bars represent the mean percentage of TCID50/mL in OSW-1 treated compared to DMSO treated cells and the error bars indicate the SEM * *p* ≤ 0.05. (D) Morphology of DMSO and OSW-1 treated BCi-ACE2 cells at 72 hpi with SARS-CoV-2. Bar = 50 μm.

To evaluate the antiviral effects of OSW-1 against SARS-CoV-2, BCi-ACE2 cells were treated with DMSO or 0.1 nM OSW-1 for 24 h before infection to induce the chemical knockdown of OSBP (Figure 4A). The cells were then washed three times to remove the DMSO/OSW-1, then infected with SARS-CoV-2 and incubated in the absence of DMSO/OSW-1 until harvested at the appropriate time point postinfection (24–72 h) for quantification of virus replication by qPCR analysis of nucleocapsid expression and production of infectious virus (Figure 4A). Compared to DMSO-treated cells, OSW-1 pretreatment significantly (*p* ≤ 0.05) decreased virus nucleocapsid expression at 24 (83.0%), 48 (62.7%), and 72 (44.6%) hpi (Figure 4B). OSW-1 pretreatment also significantly (*p* ≤ 0.05) decreased production of infectious virus at 24 (91.3%), 48 (82.4%), and 72 (80.2%) hpi compared to DMSO-treated cells (Figure 4C). Finally, comparable cell morphology was observed between SARS-CoV-2-infected DMSO and OSW-1 pretreated cells at 72 hpi, further confirming the lack of cellular toxicity associated with our OSW-1 dosing regimen (Figure 4D). In summary, our data demonstrate that OSW-1 pretreatment has antiviral activity against SARS-CoV-2 replication.

### OSW-1 Compound Treatment Increases Induction of the Host Antiviral Innate Immune Response

**Figure 5:**
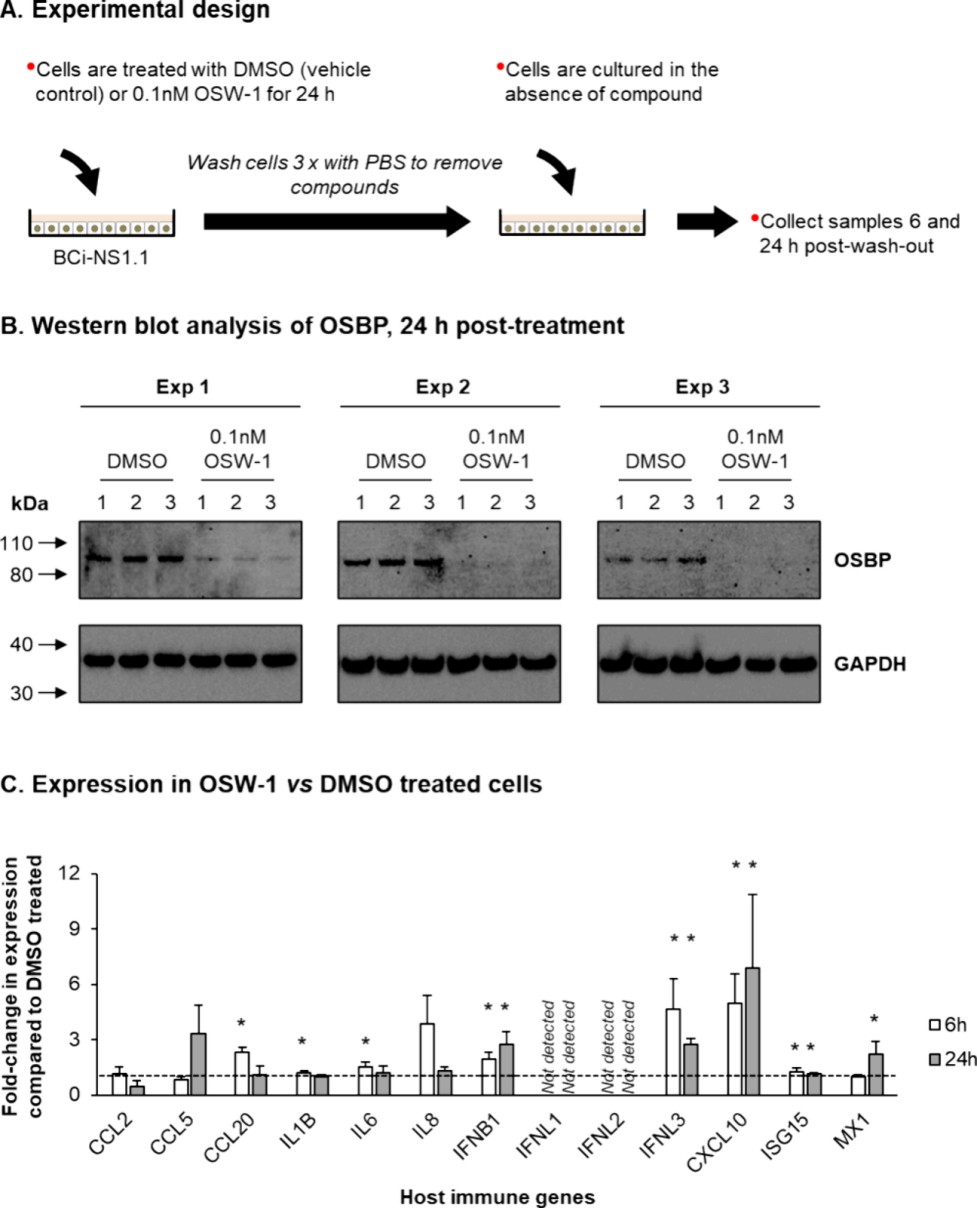
OSW-1 treatment leads to activation of the antiviral innate immune response. (A) Experimental design for the treatment of BCi-NS1.1 cells with DMSO or 0.1 nM OSW-1. (B) Western blot analysis of OSBP and GAPDH in whole cell lysates of BCi-NS1.1 cells either treated with DMSO or 0.1 nM OSW-1 for 24 h. Data shown from a *n* = 3 experiments with *n* = 3 replicates per treatment. (C) qPCR of host immune gene expression (CCL2, CCL5, CCL20, IL1B, IL6, IL8, IFNB1, CXCL10, and ISG15) at 6 (white bars) and 24 (light gray bars) hour (h) postwashout of DMSO/OSW-1. Each bar represents mean fold-change in expression compared to DMSO treated cells in *n* = 3 independent experiments and error bars indicate the SEM * *p* ≤ 0.05.

In addition to the antiviral activity, we sought to determine the effects of the OSW-1-induced chemical knockdown of OSBP on the cellular antiviral innate immune response in airway epithelial cells.^27^ Transfection of siRNA oligos to reduce protein expression is well-established to induce changes in cellular innate immunity.^33^ Our use of the OSW-1 washout treatment to induce the chemical knockdown of OSBP allows for modulating OSBP protein expression levels without the use of siRNA or related methods. Using iTRAQ proteomic analysis, we have shown previously that the OSW-1 compound washout treatment selectively reduces OSBP. Even the expression of ORP4, the protein most closely related to OSBP, is not affected by the washout OSW-1 treatment.^2^ BCi-NS1.1 cells were treated for 24 h with either DMSO or 0.1 nM OSW-1, followed by the washout of the compound-containing media. The cells were then incubated in compound-free media for either 6 or 24 h postwashout, followed by cell lysis (Figure 5A). Western blot analysis confirmed knockdown of OSBP protein levels following OSW-1 treatment (Figure 5B), and qPCR was used to assess the mRNA expression of a panel of well-known antiviral innate immune mediators in the DMSO or OSW-1 pretreated cells. Compared to DMSO pretreated cells, the OSW-1-induced chemical knockdown of OSBP led to a significant (*p* ≤ 0.05) increase in the mRNA levels of the antiviral IFN related genes IFNB1 (1.94 fold and 2.76 fold), IFNL3 (4.63 fold and 2.73 fold), CXCL10 (4.96 fold and 6.88 fold) and ISG15 (1.24 fold and 1.13 fold) at both 6 and 24 h postwashout, and MX1 (2.23 fold) at 24 h only (Figure 5C). In addition, the OSBP chemical knockdown upon OSW-1 treatment also led to a significant (*p* ≤ 0.05) increase in the expression of the proinflammatory cytokine and chemokines CCL20 (2.33-fold), IL1B (1.22-fold), and IL6 (1.53-fold) at 6 h postwashout but not at 24 h (Figure 5C). The expression levels of CCL2, CCL5, and IL8 were not changed at either 6 or 24 h, and no expression of the type III IFNs, IFNL1 and IFNL2, was detected at either time point (Figure 5C). These results suggest that the OSW-1 treatment, causing an OSBP chemical knockdown, induces the expression of multiple factors involved in mounting a cellular innate immune response, including the antiviral type I (IFNB1) and III (IFNL3) IFNs.

**Figure 6:**
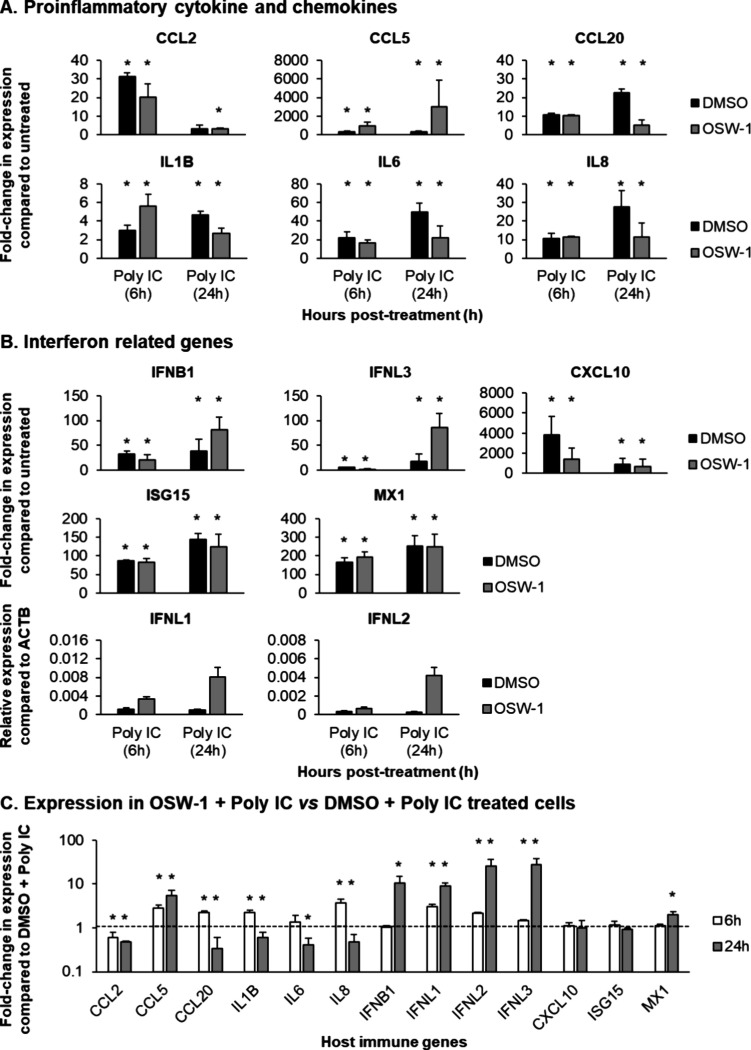
Poly IC stimulation of BCi-NS1.1 following OSW-1 treatment leads to induction of a potent innate immune response. (A) qPCR of proinflammatory cytokine and chemokine gene expression (CCL2, CCL5, CCL20, IL1B, IL6 and IL8) at 6 and 24 h (h) poststimulation with Poly IC (10 μg/mL). (B) qPCR of IFN related genes expression (IFNB1, IFNL1, IFNL2, IFNL3, CXCL10, ISG15 and MX1) at 6 and 24 h (h) poststimulation with Poly IC (10 μg/mL). For both A and B, black bars represent DMSO treated group and dark gray bars represent the OSW-1 treated group. Each bar represents mean fold-change in expression compared to untreated (non-Poly IC) cells in *n* = 3 independent experiments and error bars indicate the SEM. Due to the lack of expression of IFNL1 and IFNL2 in the untreated control group, the data are presented as relative expression compared to ACTB. * *p* ≤ 0.05 compared to the untreated control group. (C) qPCR of host immune gene expression (CCL2, CCL5, CCL20, IL1B, IL6, IL8, IFNB1, IFNL1, IFNL2, IFNL3, CXCL10, ISG15 and MX1) in OSW-1 + Poly IC treated cells at 6 (white bars) and 24 (light gray bars) hour (h) poststimulation with Poly IC (10 μg/mL). Each bar represents mean fold-change in expression compared to DMSO + Poly IC treated cells in *n* = 3 independent experiments and error bars indicate the SEM * *p* ≤ 0.05.

To better define the role of OSBP in regulating the antiviral innate immune response, we determined the effect of OSW-1 treatment in BCi-NS1.1 cells stimulated with Poly IC. Poly IC stimulation functions as a mimetic of RNA virus infection and induces a potent antiviral innate immune response without the interference of virus-expressed proteins.^34^ To identify the optimal concentration of Poly IC for induction of the antiviral innate immune response, BCi-NS1.1 cells were either untreated or treated with Poly IC (0.1, 1, and 10 μg/mL) and then harvested at 6 and 24 h post-treatment. qPCR analysis demonstrated a dose-dependent induction of multiple proinflammatory cytokines and chemokines (CCL5, CCL20, IL1B, IL6, IL8, and TNF-α) and IFN-related genes (IFNB1 and CXCL10) at both time points, with 10 μg/mL of Poly IC resulting in the most robust response (Supplementary Figure S-3). Based on these findings, a concentration of 10 μg/mL of Poly IC was used for all future experiments. To evaluate the impact of OSW-1 treatment on the Poly IC-dependent antiviral innate immune response, BCi-NS1.1 cells were treated with DMSO or 0.1 nM OSW-1 for 24 h to induce chemical knockdown of OSBP before Poly IC stimulation. The cells were washed three times to remove the DMSO/OSW-1, then either untreated or treated with Poly IC (10 μg/mL) and harvested at 6 and 24 h post-treatment. In both DMSO and OSW-1 treated cells, Poly IC stimulation led to a significant (*p* ≤ 0.05) induction in expression of the proinflammatory cytokines and chemokines CCL2 (31.2 fold DMSO, 20.2 fold OSW-1), CCL5 (287.8 fold DMSO, 954.1 fold OSW-1), CCL20 (10.7 fold DMSO, 10.5 fold OSW-1), IL1B (3.0 fold DMSO, 5.6 fold OSW-1), IL6 (22.2 fold DMSO, 16.3 fold OSW-1) and IL8 (10.5 fold DMSO, 11.4 fold OSW-1) at 6 h post-treatment (Figure 6A). Furthermore, a significant (*p* ≤ 0.05) Poly IC-dependent induction of the same genes (CCL2 (3.3 fold OSW-1), CCL5 (337.0 fold DMSO, 3031.0 fold OSW-1), CCL20 (22.4 fold DMSO, 5.1 fold OSW-1), IL1B (4.7 fold DMSO, 2.7 fold OSW-1), IL6 (49.4 fold DMSO, 21.6 fold OSW-1) and IL8 (27.6 fold DMSO, 11.5 fold OSW-1)) was still observed in both DMSO (except for CCL2) and OSW-1 treated cells at 24 h post-treatment (Figure 6A). Analysis of the IFN related genes revealed a similar trend, with Poly IC stimulation leading to a significant (*p* ≤ 0.05) induction in expression of all genes at 6 h (IFNB1 (32.6 fold DMSO, 21.3 fold OSW-1), IFNL3 (5.6 fold DMSO, 2.18 fold OSW-1), CXCL10 (3863.8 fold DMSO, 1434.4 fold OSW-1), ISG15 (86.2 fold DMSO, 82.8 fold OSW-1) and MX1 (166.7 fold DMSO, 194.5 fold OSW-1)) and 24 h (IFNB1 (38.0 fold DMSO, 81.9 fold OSW-1), IFNL3 (18.5 fold DMSO, 86.1 fold OSW-1), CXCL10 (891.2 fold DMSO, 702.7 fold OSW-1), ISG15 (144.6 fold DMSO, 124.0 fold OSW-1) and MX1 (254.5 fold DMSO, 248.8 fold OSW-1)) post-treatment (Figure 6B). Despite the lack of detectable expression of IFNL1 and IFNL2 in non-Poly IC stimulated cells, stimulation of both DMSO and OSW-1 treated cells with Poly IC led to induction of both genes at 6 and 24 h post-treatment (Figure 6B).

To investigate potential additive effects of OSW-1 treatment on Poly IC-dependent innate immune activation, we compared the expression levels of each immune gene (CCL2, CCL5, CCL20, IL1B, IL6, IL8, IFNB1, IFNL1, IFNL2, IFNL3, CXCL10, ISG15 and MX1) in OSW-1 + Poly IC vs DMSO + Poly IC treated cells (Figure 6C). At 6 h post-treatment, a significant (*p* ≤ 0.05) reduction in the expression of CCL2 (0.60 fold) and an increase in the expression of CCL5 (2.84 fold), CCL20 (2.28 fold), IL1B (2.25 fold), IL8 (3.77 fold), IFNL1 (3.05 fold), IFNL2 (2.15 fold) and IFNL3 (1.46 fold) was observed in OSW-1 + Poly IC vs DMSO + Poly IC treated cells (Figure 6C). No significant (*p* ≤ 0.05) differences in the expression of IL6, IFNB1, CXCL10, ISG15, and MX1 were observed at this time point (Figure 6C). However, at 24 h post-treatment, a significant (*p* ≤ 0.05) reduction in the expression of CCL2 (0.47 fold), CCL20 (0.34 fold), IL1B (0.61 fold), IL6 (0.41 fold) and IL8 (3.77 fold) and an increase in the expression of CCL5 (5.41 fold), IFNB1 (10.59 fold), IFNL1 (9.12 fold), IFNL2 (25.36 fold), IFNL3 (27.25 fold) and MX1 (1.98 fold) was observed in OSW-1 + Poly IC vs DMSO + Poly IC treated cells (Figure 6). Similar to the 6 h time point, no significant (*p* ≤ 0.05) differences in the expression of CXCL10 and ISG15 were observed (Figure 6C). Combined, these data suggest that OSW-1 pretreatment leads to enhanced expression of proinflammatory cytokines and chemokines (CCL5, CCL20, IL1B, and IL8) at early stages (6 h) postinnate immune activation with Poly IC treatment, which despite maintaining higher levels of CCL5, then reduces at late stages (24 h) and switches to enhanced production of IFNB1, IFNL1, IFNL2, IFNL3 and MX1. The 9–27 fold increase in IFNB1, IFNL1, IFNL2, and IFNL3 mRNA expression in the OSW-1 treated Poly-IC stimulated cells suggests that the loss of OSBP protein significantly ramps up type I and III IFN responses.

## Discussion

Building on our previous work, we demonstrate that OSBP is a host factor that plays an important role in the replication of multiple pathogenic (+)ssRNA viruses. The targeting of OSBP via OSW-1 compound treatment inhibits the replication of the human rhinovirus (HRV1B) and multiple coronaviruses that infect humans (HCoV-229E and SARS-CoV-2) and animals (FIPV). In addition, we demonstrate that OSW-1 displays a more potent antiviral activity against SARS-CoV-2 compared to other compounds (e.g., ITZ, THEV, and TTP) reported to function through targeting OSBP.^3,5,7,8,16,19^ In support of our findings, a recent publication demonstrated that an OSW-1 analog compound inhibits coronavirus replication (including SARS-CoV-2) in human hepatoma (Huh-7) and human colorectal adenocarcinoma (Caco-2) cells via targeting of OSBP.^35^ Combined, these studies suggest that OSBP is a potential host target for the development of novel broad-spectrum (+)ssRNA antiviral therapeutics. Based on its function as a lipid transporter, prior studies have postulated that OSBP regulates the replication of multiple (+)ssRNA viruses via aiding the formation of membrane-wrapped VRO that are required to evade the host antiviral response and promote replication.^17^ Therefore, hypothetically, the OSW-1-compound-induced loss of OSBP expression could inhibit VRO formation or VRO integrity, which could limit viral replication. In support of this, recent work by Ma-Lauer et al. presented results suggesting that OSBP interacts with multiple SARS-CoV-2 proteins involved in VRO formation.^35^ However, our results show that OSW-1 treatment activates the components of the antiviral innate immune system in airway epithelial cells, implicating a potential antiviral role of OSBP beyond its role in VRO formation. OSW-1 chemical knockdown of OSBP in the airway epithelial cells enhances the activation of specific components of innate immunity, especially elements of type I and III IFN responses, following stimulation with the viral mimetic Poly IC.

Interferons are a multigene family of proteins that play a crucial role in regulating the host antiviral response to virus infection.^36,37^ The IFNs are classified into three types (I–III), with the type I (e.g., IFNB1) and type III (e.g., IFNL1–3) functioning as the major antiviral IFNs.^36−38^ Expression of IFNs is induced in response to virus infection by multiple cytoplasmic nucleic acid sensors, including the cytosolic RIG-I-like receptors (RLRs) and endosomal Toll-like receptors (TLRs), which recognize invading virus genomes or replication intermediates (i.e., single-stranded and double-stranded RNA).^36,37^ The sensing of virus-derived nucleic acids leads to activation of multiple downstream signaling cascades and transcription factors (e.g., IRF3, IRF7, and NFkB) which induce IFN expression.^36,37^ In turn, secreted IFNs exert their antiviral activities in an autocrine or paracrine manner by binding to type-specific receptors on the surface of cells and triggering multiple signaling cascades to induce expression of IFN-stimulated genes (ISGs) (e.g., CXCL10, ISG15, MX1) that function to restrict virus replication.^36,37^ Our findings demonstrate that OSW-1 treatment of human airway epithelial cells: (1) leads to induction of multiple IFN related genes (IFNB1, IFNL3, CXCL10, ISG15 and MX1); and (2) enhances the activation of specific components of the type I (IFNB1) and III (IFNL1, IFNL2 and IFNL3) IFN antiviral response following stimulation with Poly IC. Therefore, these data suggest that the broad spectrum antiviral activity of OSW-1 treatment against multiple (+)ssRNA viruses (i.e., HRV1B, HCoV-229E, FIPV, and SARS-CoV-2) may occur via activation of the IFN-dependent host antiviral defenses. In support of this, studies have demonstrated that treatment of cells with type I and III IFNs can suppress HRV1B,^39^ HCoV-229E,^39^ FIPV,^40^ and SARS-CoV-2 replication.^41−43^ In addition to activation of type I and type III IFN signaling, our data demonstrate that OSW-1 treatment leads to reduced levels of proinflammatory cytokines and chemokines (CCL2, CCL20, IL1B, IL6, and IL8) at late stages (i.e., 24 h) poststimulation with Poly IC. In the context of SARS-CoV-2 infection, an exaggerated proinflammatory response (i.e., cytokine storm) is associated with severe COVID-19 and lung injury.^44,45^ Therefore, in addition to its antiviral activities, targeting of OSBP function (e.g., via OSW-1 treatment) may also reduce disease severity and lung damage by suppressing the induction of the host cytokine storm in response to virus infection. However, future studies using in vivo animal models of virus infection (e.g., mouse-adapted strains of SARS-CoV-2) are required to investigate this further.

Our study builds on our previous findings and demonstrates a new role for OSBP as a likely regulator of the antiviral innate immune response. ssRNA viral replication organelles and elements of the innate immune system have been shown to have an enmeshed and complex interplay.^46^ As shown, the OSW-1-chemical knockdown of OSBP protein levels alters the expression of cellular innate immune factors, including antiviral type I and III IFNs, even without the Poly IC stimulation. This suggests that loss of OSBP function primes or triggers expression of the observed innate immune factors, which suggests that this OSBP-centered effect is independent of the formation of the VRO.^17^ The role of the OSBP in regulating the antiviral innate immune response is supported by a recent paper reporting that itraconazole, which is an antifungal drug that inhibits OSBP in addition to several other proteins, increases PI4P abundance and activates the cyclic GMP-AMP synthase-stimulator of interferon genes (cGAS-STING) pathway.^47^ The cGAS-STING pathway activates the expression of IFNs and other antiviral elements upon detection of cytosolic DNA and in response to RNA virus infection.^48^ Therefore, future research will be required to investigate the role of the cGAS-STING pathway in regulating the effects on OSBP loss (due to OSW-1 compound treatment) and activation of the antiviral innate immune response in the absence and presence of Poly IC or upon (+)ssRNA virus infection.

In summary, our results suggest that the OSW-1 compound may have a dual antiviral mechanism of action through targeting of OSBP. First, OSBP loss of function limits viral replication through preventing VRO formation. And second, OSBP loss of function enhances the antiviral innate immune response. Both parts of the dual mechanism contribute to the prophylactic antiviral activity of the OSW-1 compound. However, we acknowledge that there are some limitations with our study. The OSW-1 compound is a well-established, selective chemical probe to study OSBP and ORP4, but further studies using methods to alter the OSBP expression and function will be useful in confirming our observed OSW-1 effect. Since transfection with siRNA and related knockdown methods alter cellular innate immunity,^33^ other methods such as CRISPR Cas9-mediated gene knockout or other classes of OSBP-targeting small molecules could be used. Future studies studying the effects of OSBP on innate antiviral immunity upon viral infection, as opposed to Poly IC, will also be important. Despite these limitations, our findings support further investigation into the role of OSBP in mediating the antiviral innate immune response as well as efforts to develop novel broad-spectrum (+)ssRNA antiviral therapeutics through targeting OSBP.

## Experimental Section

### Isolation of OSW-1 Compound from Natural Sources

The OSW-1 compound was isolated from the *Ornithogalum saundersiae* bulbs as previously described.^19^ Stocks of OSW-1 were made in dimethyl sulfoxide (DMSO, catalog number 3512–12, Sigma-Aldrich, St. Louis, MO, USA) and stored at −20 °C. >95% purity of OSW-1 was determined via HPLC analysis (Supplementary Figure S-1). ITZ was purchased from Sigma-Aldrich (catalog I6657) as a 1:1:1:1 mixture of diastereomers. Both the T-00127-HEV2 (THEV) and TTP-8307 (TTP) compounds were synthesized in our group, as reported previously.^3^

### Generation and Titration of HRV-1B, HCoV-229E, FIPV, and SARS-CoV-2 Stocks

The virus strains HRV1B (ATCC VR-1645), HCoV-229E (ATCC VR-740), and FIPV (strain: WSU 79–1146, catalog number ATCC VR-990) were all purchased from the American Type Culture Collection (ATCC) (Manassas, VA, USA), whereas the SARS-CoV-2 Washington strain (isolate USA-WA1/2020) was obtained from BEI Resources (catalog number NR-52281, Manassas, VA, USA). Virus stocks were generated using H1-HeLa (HRV-1B), MRC-5 human lung fibroblasts (HCoV-229E), Crandall-Reese Feline kidney (CRFK) cells (FIPV), and Vero-E6 (SARS-CoV-2) as previously described.^16,49^ H1-HeLa and Vero-E6 cells were grown in DMEM High Glucose media (catalog number 11965092, Thermo Fisher Scientific, Waltham, MA, USA) + 10% fetal bovine serum (FBS) (catalog number S11550, R&D Systems, Inc., Minneapolis, MN, USA) with 1% Pen/Strep (10,000 U/mL) (catalog number 15140122, Thermo Fisher Scientific). MRC-5 cells were grown in MEM (catalog number 10010CM, Corning, Corning, NY, USA) + 10% FBS (catalog number S11550, R&D Systems, Inc.) + 2 mM l-Glutamine (catalog number 25030081, Thermo Fisher Scientific) + 1% Pen/Strep (10,000 U/mL) (catalog number 15140122, Thermo Fisher Scientific). The titer of each virus stock was then calculated on the following cell lines using the 50% tissue culture infectious dose (TCID_50_) method, as previously described: FIPV and HRV1 on H1 HeLa cells, HCoV-229E on MRC-5, and SARS-CoV-2 on Vero E6.^49^ All of the experiments involving SARS-CoV-2 were performed in the High Containment Biosafety Level-3 Laboratory Core at either Oklahoma State University (OSU) or the University of Oklahoma Health Sciences Center (OUHSC), according to the guidelines approved by the Institutional Biosafety Committee at each institution.

### Testing the Antiviral Effects of OSW-1 Treatment against HRV1B, HCoV-229E, FIPV, and SARS-CoV-2

To investigate the antiviral effects of OSW-1 treatment on HRV1B, HCoV-229E, FIPV, and SARS-CoV-2 replication, experiments were performed in H1-HeLa, MRC-5, CRFK, and Vero E6 cells, respectively. Viral inhibition assays were performed in 24-well plates. One × 10^5^ cells/well of H1-HeLa cells were seeded for HRV1B. Five × 10^5^ cells/well of MRC-5 cells were seeded for HCoV-229E. One × 10^5^ cells/well of CRFK were seeded for FIPV; 1 × 10^5^ cells/well of VeroE6 cells were seeded for SARS-CoV-2. After overnight incubation, the plated cells were treated with either DMSO (vehicle control) or compound (i.e., OSW-1, ITZ, THEV or TTP) at the indicated concentrations for 6 h. Then, the compound-containing media were removed, and the cells were then infected with each virus at a multiplicity of infection (MOI) of 1.0 in 0.5 mL of viral inoculum for 30 min at 37 °C. Following infection, the virus inoculum was removed, and the cells were washed with 1 mL of complete media. Then, fresh media containing either DMSO or compound at the indicated concentrations was added back to the cells for a 10 h incubation, before harvest. At the time of harvest, the cells were frozen at −80 °C and the TCID_50_ values calculated as described above. For each independent experiment, experimental treatments were assessed in *n* = 4 replicates with the means used for statistics.

### Culture of BCi-NS1.1 Cells

BCi-NS1.1 cells were maintained in BronchiaLife epithelial airway medium (BLEAM) (catalog number LL-0023; Lifeline Cell Technology, Frederick, MD, USA) supplemented with 1% Pen/Strep (10,000 U/mL) (catalog number 15140122, Thermo Fisher Scientific) as described previously for primary human bronchial epithelial cells.^49^ Generation of a BCi-NS1.1 cell line overexpressing human ACE2 (BCi-ACE2) was previously described in detail.^32^ Both BCi-Control and BCi-ACE2 cells were maintained in a manner identical to that of the parental BCi-NS1.1 cells.

### Cytotoxicity Analysis of OSW-1 in BCi-NS1.1 Cells

The cytotoxicity of OSW-1 in BCi-NS1.1 cells was assessed using the CellTiter-Blue assay. For each independent experiment, experimental treatments were assessed in *n* = 3 replicates with the means used for statistics.

### RNA Extraction, cDNA Synthesis, and qPCR Gene Expression Analysis

Total RNA was extracted via direct lysis of cells in the culture plate (following removal of the culture media) using the PureLink RNA mini kit (catalog number 12183018A, Thermo Fisher Scientific). To remove contaminating genomic DNA, DNase treatment (catalog number 12185–010, Thermo Fisher Scientific) was applied on the column. Complementary DNA (cDNA) was generated from an equal amount of total RNA per sample using random hexamers (Applied Biosystems High Capacity cDNA Reverse Transcription Kit, catalog no. 4374966, Thermo Fisher Scientific). Quantitative PCR (qPCR) was performed as previously described.^32^ The relative expression levels of specific genes were analyzed in duplicate and determined using the dCt method with actin beta (ACTB) as the endogenous control. PrimePCR gene-specific primers were purchased from Bio-Rad (Hercules, CA, USA), and the assays were performed using the manufacturer’s recommended cycling parameters. Specific primers used are listed in the Supporting Information. Expression of the SARS-CoV-2 nucleocapsid gene was quantified using the Centers for Disease Control (CDC) designed primers nCOV_N1 Forward Primer (catalog number 10006821) and nCOV_N1 Reverse Primer (catalog number 10006822) purchased from IDT (San Diego, CA, USA) as described previously.^32^ For each time point and condition, the gene expression levels were assessed in *n* = 3 replicates with the means used for statistics.

### Western Blot Analysis

The following primary antibodies were used for Western blot analysis: OSBP (1:1000 dilution, catalog number sc-365771, Santa Cruz Biotechnology Inc., Dallas, TX, USA), GAPDH (1:5000 dilution, catalog number 2118S, Cell Signaling Technologies, Danvers, MA, USA) and ACE2 (1:1000 dilution, catalog number NBP2–67692, Novus Biologicals, Centennial, CO, USA). The abundance of OSBP (relative to GAPDH levels) was quantified using ImageJ software (version 1.8.0_112, NIH).

### Immunofluorescence Staining of the SARS-CoV-2 Nucleocapsid

BCi-Control and BCi-ACE2 cells (5 × 10^4^) were seeded into chamber slides (catalog number 354114, Corning) in 1 mL of BLEAM. The next day, the cells were infected with SARS-CoV-2 in 0.5 mL of BLEAM at an MOI of 0.1 for 2 h at 37 °C. Following infection, the virus inoculum was removed, and the cells were washed three times with 1 mL of phosphate-buffered saline (PBS) (catalog number 10010023, Thermo Fisher Scientific), then incubated in 1 mL of BLEAM. At 48 h postinfection, the media was removed, and the cells were fixed with 10% neutral buffered formalin (catalog number 51201, Expredia, Kalamazoo, MI, USA) for 20 min at room temperature (RT). Following fixation, the cells were permeabilized with 0.1% Triton-X 100 (catalog number 194854, MP Biomedicals, Irvine, CA, USA) for 10 min at RT, followed by blocking with 10% goat serum (catalog number 0929391-CF, MP Biomedicals) for 30 min at RT. Once blocked, the cells were incubated with a primary antibody against SARS-CoV-2 nucleocapsid (10 μg/mL, catalog number MA1–7403, Thermo Fisher Scientific) for 2 h at RT and then washed three times with PBS, followed by incubation with a fluorescently labeled secondary antibody (2 μg/mL, catalog number A11029, Goat antimouse Alexa Fluor 488, Thermo Fisher Scientific) for 1 h at RT. The cell nuclei were counterstained with DAPI (1 μg/mL, catalog number 62248, Thermo Fisher Scientific). Images were taken using an Olympus BX43 upright fluorescent microscope (Olympus Corporation, Tokyo, Japan).

### Analysis of SARS-CoV-2 Replication Kinetics in BCi-ACE2 Cells

BCi-ACE2 cells (1 × 10^5^) were seeded into each well of a 12-well plate (catalog number 3513, Corning) in 1 mL of BLEAM. The next day, the cells were either uninfected (mock) or infected with SARS-CoV-2 in 0.5 mL of BLEAM at an MOI of 0.1 for 2 h at 37 °C. Following infection, the virus inoculum was removed, and the cells were washed three times with 1 mL of PBS, then incubated in 1 mL of BLEAM. At each time point postinfection (24–72 h), mock- or SARS-CoV-2-infected cells were collected for RNA extraction and the media for quantification of virus production by TCID_50_ assay using Vero E6-TMPRSS2-T2A-ACE2 cells (catalog number NR-54970, BEI Resources) as previously described.^32^ For each independent experiment, experimental conditions and time points were assessed in *n* = 3 replicates with the means used for statistics.

### Testing the Antiviral Effects of OSW-1 Treatment against SARS-CoV-2

For experiments investigating the antiviral effects of OSW-1 treatment on SARS-CoV-2 replication, BCi-ACE2 cells (1 × 10^5^) were seeded in an identical manner described above and the next day treated for 24 h with either DMSO (vehicle control) or 0.1 nM OSW-1. Following treatment, the cells were washed three times with 1 mL of PBS to remove the DMSO/OSW-1, then infected with SARS-CoV-2 at a MOI of 0.1 and harvested at the appropriate time point postinfection (24–72 h) for quantification of virus replication by qPCR analysis of nucleocapsid expression and production of infectious virus as described above. For each independent experiment, experimental conditions and time points were assessed in *n* = 3 replicates with the means used for statistics.

### Stimulation of Cells with Poly IC

BCi-NS1.1 cells (1 × 10^5^) were seeded into each well of a 12-well plate (catalog number 3513, Corning) in 1 mL of BLEAM. The next day the media was replaced with 1 mL of fresh BLEAM (untreated) or BLEAM supplemented with differing concentrations (0.1, 1, or 10 μg/mL) of poly IC (catalog number tlrl-pic, InvivoGen, San Diego, CA, USA), and harvested for analysis at 6 and 24 h post-treatment. For each independent experiment, experimental conditions and time points were assessed in *n* = 3 replicates with the means used for statistics.

### Statistics

Statistical analysis of comparisons between groups (i.e., OSW-1 vs DMSO-treated controls or SARS-CoV-2-infected vs uninfected controls) was performed using SPSS Version 27.0 software (SPSS Inc., Chicago, IL, USA) with statistical significance determined as a *p* value of ≤0.05 (*p* ≤ 0.05) using the Mann–Whitney *U* test.

## Supporting Information Available

Additional supporting figures and detailed experimental methods, OSW-1 purity and characterization data, OSBP and ORP4 expression levels in BCi-NS1.1 cells, including upon OSW-1 treatment, and effect of Poly IC stimulation on innate immune factors in BCi-NS1.1 cells (PDF)

## Dedication

^○^ In memoriam 6/14/2022.

## Abbreviations

BLEAM: BronchiaLife epithelial airway medium
cDNA: complementary DNA
cGAS-STING: cyclic GMP-AMP Synthase-Stimulator of Interferon Genes
COVID-19: coronavirus disease 2019
CRFK cells: Crandall-Reese Feline Kidney cells
DMSO: dimethyl sulfoxide
ER: endoplasmic reticulum
FDA: Food and Drug Administration
FIPV: feline infectious peritonitis virus
GI_50_: 50% cell growth inhibition
HCoV-229E: human coronavirus 229E
HRV1B: human rhinovirus 1B
IFN: interferon
ORPs: OSBP-related proteins
OSBP: oxysterol-binding protein
PBS: phosphate buffered saline
PI4P: phosphoinositide-4-phosphate
Poly IC: polyinosinic-polycytidylic acid
qPCR: quantitative polymerase chain reaction
(+)ssRNA: positive-sense (+) single-stranded RNA (ssRNA)
RSV: respiratory syncytial virus
RT: room temperature
SARS-CoV-2: severe acute respiratory syndrome coronavirus 2
SD: standard deviation
SEM: standard error of the mean
TCID_50_: 50% tissue culture infectious dose
VROs: viral replication organelles
