# Supplementary material for: Anti-SARS-CoV-2 Small Molecule Targeting of Oxysterol-Binding Protein (OSBP) Activates Cellular Antiviral Innate Immunity
Source: ACS Infect Dis. 2025 Apr 21;11(5):1064–77. doi: 10.1021/acsinfecdis.4c00631 (PMC12070403; doi:10.1021/acsinfecdis.4c00631)
Supplement: Supplementary file 1 — id4c00631_si_001.pdf [file id4c00631_si_001.pdf]

SUPPORTING INFORMATION

Anti-SARS-CoV-2 Small Molecule Targeting of Oxysterol-Binding Protein (OSBP) Activates Cellular Antiviral Innate Immunity

Bharathiraja Subramaniyan [a], Emily C. Falcon [b,$], Andrew R. Moore [a], Jason L. Larabee [c], Susan L. Nimmo [b], Jorge L. Berrios-Rivera [b], William J. Reddig [d], Earl L. Blewett [d,†], James F. Papin [e,*], Matthew S. Walters [a,*], Anthony W. G. Burgett [b,f,*]

SUPPLEMENTARY RESULTS

**Figure S-1**	**2**
**Figure S-2**	**3**
**Figure S-3**	**4-5**

SUPPLEMENTARY METHODS **6-12**

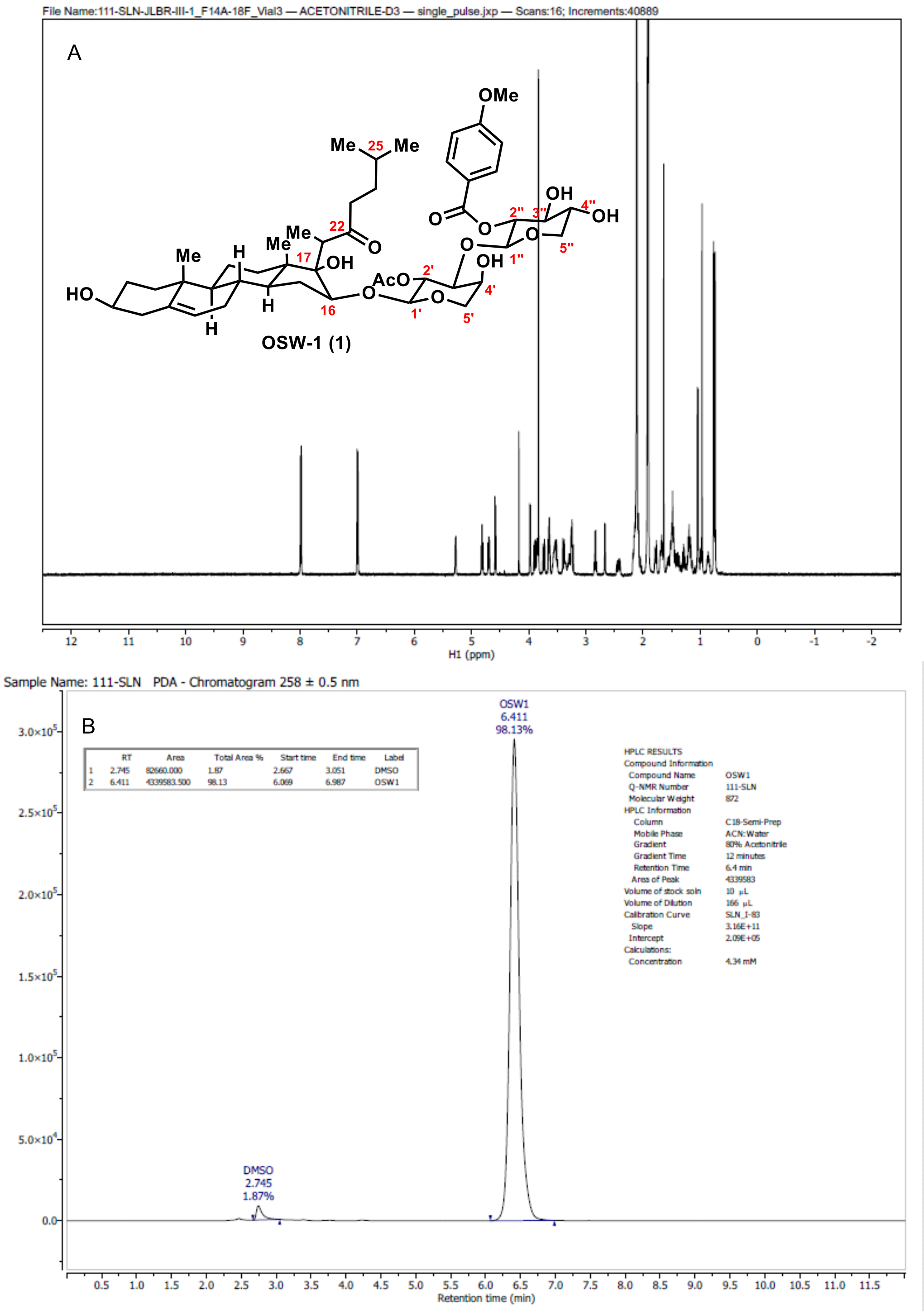

Figure S-1: (A) ^{1}H-NMR of HPLC purified OSW-1. **(B)** Reverse phase HPLC analysis of 10 mM OSW-1 stock solution in DMSO, used for biological experiments.

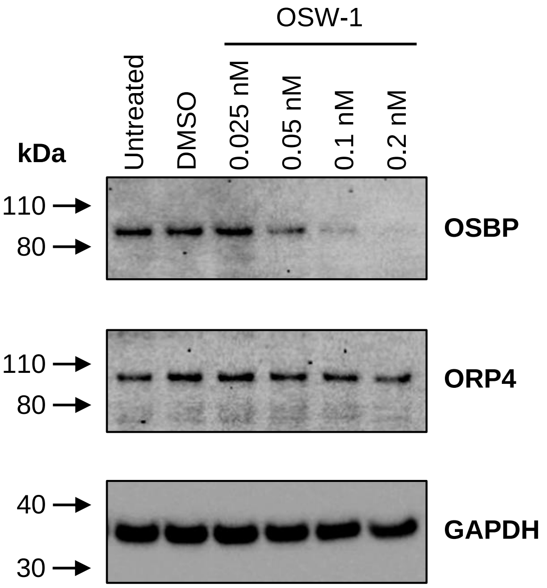

Figure S-2: Treatment of BCi-NS1.1 cells with OSW-1 selectively reduces OSBP protein levels. Western blot analysis of OSBP, ORP4 and GAPDH in whole cell lysates of BCi-NS1.1 cells either untreated or treated with DMSO or varying concentrations of OSW-1 (0.025, 0.05, 0.1 and 0.2 nM) for 24 h.

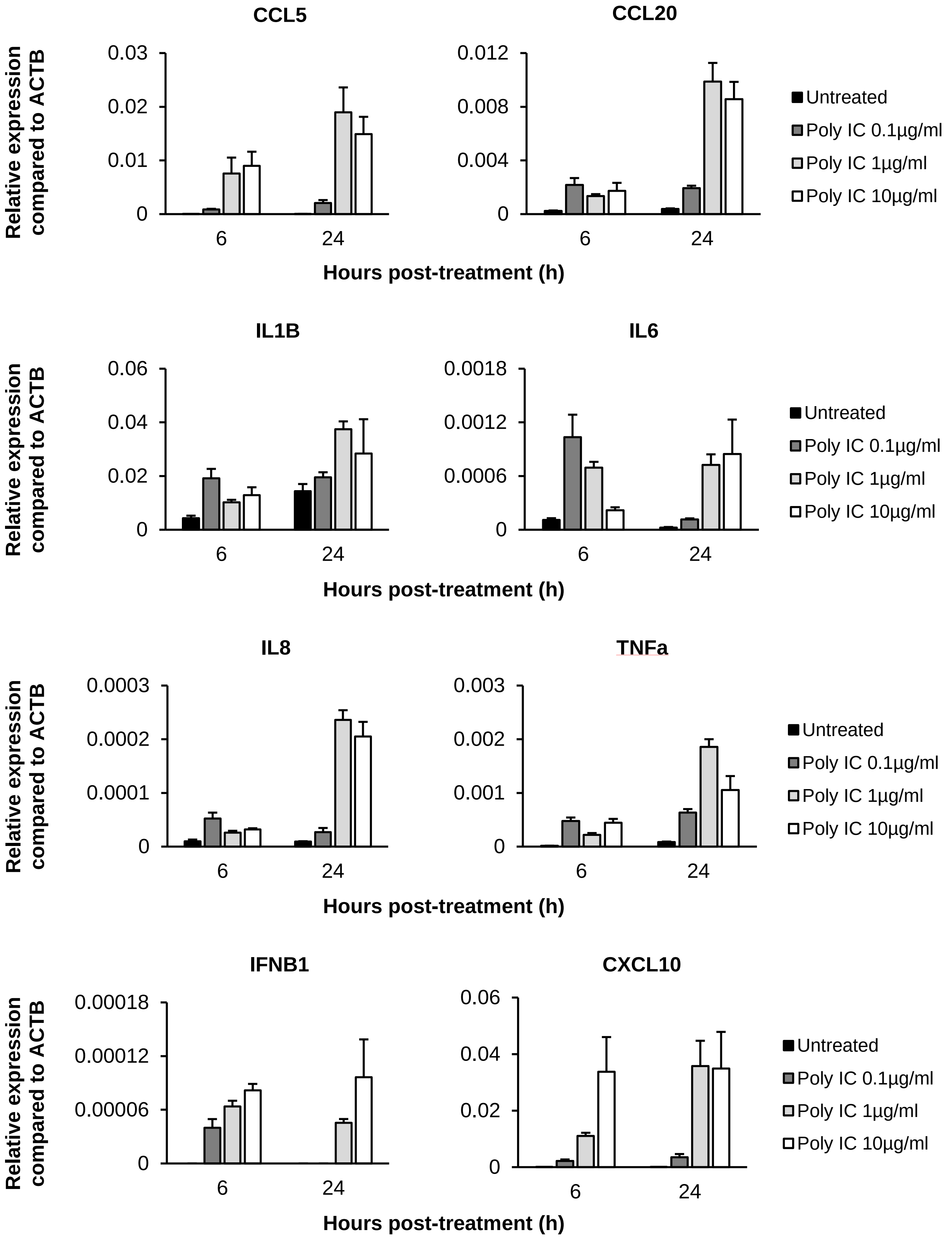

CCL5
CCL20
IL1B
IL6
IL8
TNFa
IFNB1
CXCL10
Relative expression compared to ACTB
Hours post-treatment (h)
6
24
0
0.01
0.02
0.03
0.004
0.008
0.012
0.04
0.06
0.0006
0.0012
0.0018
0.0001
0.0002
0.0003
0.001
0.002
0.003
0.00006
0.00012
0.00018
Untreated
Poly IC 0.1µg/ml
Poly IC 1µg/ml
Poly IC 10µg/ml

Figure S-3: Poly IC stimulation of BCi-NS1.1 induces a potent innate immune response. BCi-NS1.1 cells were either untreated or treated with multiple concentrations (0.1, 1 and 10 μg/mL) of Poly IC and harvested 6 and 24 hours (h) post-stimulation for qPCR analysis of host immune gene expression (CCL5, CCL20, IL1B, IL6, IL8, TNF-α, IFNB1 and CXCL10). Bars represent mean expression in n=3 replicates from a single experiment and the error bars indicate the SEM.

SUPPLEMENTARY METHODS

Isolation of OSW-1 compound from natural source

Ornithogalum saundersiae bulbs were grown at the University of Oklahoma greenhouse in Norman, OK. OSW-1 compound was isolated from the bulbs and purified (Supplementary Figure 1) as similar to previously described. [21] Stocks of OSW-1 were made in dimethyl sulfoxide (DMSO, catalog number 3512-12, Sigma-Aldrich, St. Louis, MO, USA) and stored at -20°C.

Generation and titration of HRV-1B, HCoV-229E, FIPV and SARS-CoV-2 stocks

The virus strains HRV1B (ATCC VR-1645), HCoV-229E (ATCC VR-740), and FIPV (strain: WSU 79-1146, catalog number ATCC VR-990) were all purchased from the American Type Culture Collection (ATCC) (Manassas, VA, USA), whereas SARS-CoV-2 Washington strain (isolate USA-WA1/2020) was obtained from BEI Resources (catalog number NR-52281, Manassas, VA, USA). Virus stocks were generated using H1-HeLa (HRV-1B), MRC-5 human lung fibroblasts (HCoV-229E), Crandall-Reese Feline Kidney (CRFK) cells (FIPV) and Vero-E6 (SARS-CoV-2) as previously described.[17,51] H1-HeLa and Vero-E6 cells were grown in DMEM High Glucose media (catalog number 11965092, Thermo Fisher Scientific, Waltham, MA, USA) + 10% fetal bovine serum (FBS) (catalog number S11550, R&D Systems, Inc., Minneapolis, MN, USA) with 1% Pen/Strep (10,000 U/mL) (catalog number 15140122, Thermo Fisher Scientific). MRC-5 cells were grown in MEM (catalog number 10010CM, Corning®, Corning, NY, USA) + 10% FBS (catalog number S11550, R&D Systems, Inc.) + 2 mM L-Glutamine (catalog number 25030081, Thermo Fisher Scientific) + 1% Pen/Strep (10,000 U/mL) (catalog number 15140122, Thermo Fisher Scientific). VeroE6 cells were cultured in EMEM, 10% FBS with L-glutamine and 1% Pen/Strep. The titer of each virus stock was then calculated on the following cell lines using the 50% tissue culture infectious dose ($TCID_{50}$) method as previously described: FIPV and HRV1

on H1 HeLa cells, HCoV-229E on MRC-5, and SARS-CoV-2 on Vero E6.[51] All the experiments involving SARS-CoV-2 were performed in the High Containment Biosafety Level-3 Laboratory Core at the University of Oklahoma Health Sciences Center (OUHSC), according to the guidelines approved by the Institutional Biosafety Committee.

Testing the antiviral effects of OSW-1 treatment against HRV1B, HCoV-229E and FIPV

To investigate the antiviral effects of OSW-1 treatment on HRV1B, HCoV-229E and FIPV replication, experiments were performed in H1-HeLa, MRC-5 and CRFK cells respectively. All cell culture experiments with these cell lines were performed in a humidified atmosphere with 5% CO_2 at 37°C. Viral inhibition assays were performed in 24-well plates. 1 x 10^5 cells/well of H1-HeLa cells were seeded for HRV1B. 1 x 10^5 cells/well of CRFK were seeded for FIPV. 5 x 10^5 MRC-5 cells/well were seeded for HCoV-229E. The next day, cells were treated with either DMSO (vehicle control) or OSW-1 (1, 10, and 30 nM) for 6 hours prior to infection. The media was removed, and the cells were then infected with each virus at a multiplicity of infection (MOI) of 1.0 in 0.5 mL of viral inoculum for 30 minutes at 37°C. Following infection, the virus inoculum was removed and the cells washed with 1 mL of media. Then, fresh media with either DMSO or OSW-1 was added back to the cells for 10 hours before harvest. At the time of harvest, the cells were frozen at -80°C and the $TCID_{50}$ values calculated as described above. For each independent experiment, experimental treatments were assessed in n=4 replicates with the means used for statistics.

Culture of BCi-NS1.1 cells

BCi-NS1.1 cells were maintained in BronchiaLife™ epithelial airway medium (BLEAM) (catalog number LL-0023; Lifeline Cell Technology, Frederick, MD, USA) supplemented with 1% Pen/Strep (10,000 U/mL) (catalog number 15140122, Thermo Fisher Scientific) as described

previously for primary human bronchial epithelial cells.[51] All cell culture experiments with BCi-NS1.1 cells were performed in a humidified atmosphere with 5% CO_2 at 37°C. Generation of a BCi-NS1.1 cell line over-expressing human ACE2 (BCi-ACE2) was previously described in detail. [35] Briefly, BCi-NS1.1 cells were infected with a replication-deficient lentivirus expressing human ACE2 under control of the eukaryotic translation elongation factor 1 α (EF-1α) promoter (catalog number EX-U1285-Lv160, GeneCopoeia Inc, Rockville, MD, USA) and subsequently treated with 500 µg/mL of G418/Neomycin (catalog number 30-234-CR, Corning®, Corning, NY, USA) for 2 weeks to select for transduced cells and establish a stable cell line constitutively over-expressing human ACE2. BCi-Control cells were created in tandem via infection with the empty vector control lentivirus (catalog number EX-NEG-Lv160, GeneCopoeia Inc) that lacks expression of human ACE2. Both BCi-Control and BCi-ACE2 cells were maintained in an identical manner to the parental BCi-NS1.1 cells.

Cytotoxicity analysis of OSW-1 in BCi-NS1.1 cells

The cytotoxicity of OSW-1 in BCi-NS1.1 cells was assessed using the CellTiter-Blue™ assay (catalog number PRG8081, Promega, Madison, WI, USA). Briefly, BCi-NS1.1 cells (average 3500) were seeded into 96-well plates (catalog number 3916, Corning®) in 100 µl of BLEAM, and the next day treated in triplicate with serial dilutions of DMSO (vehicle control), OSW-1 or the positive control compound Taxol® (paclitaxel) (catalog number S1150, Selleck Chemicals LLC, Houston, TX, USA) in BLEAM for 24-72 hours. At each time point, the cells were incubated with CellTiter-Blue™ reagent for 1-2 hours and then read at 544 excitation/590 emission using a Spectramax M3 plate reader (Molecular Devices. San Jose, CA, USA). The percentage of live cells was calculated using a background subtraction of time zero untreated cells with Cell-Titer Blue reagent and normalizing to the DMSO control wells. Cell counts were graphed versus

concentration, and an inhibition dose-response curve was calculated in GraphPad Prism. The GI_{50} (50% cell growth inhibition) value was calculated from the dose-response curve in GraphPad Prism. For each independent experiment, experimental treatments were assessed in n=3 replicates with the means used for statistics.

RNA extraction, cDNA synthesis and qPCR gene expression analysis

Total RNA was extracted via direct lysis of cells in the culture plate (following removal of the culture media) using the PureLink™ RNA mini kit (catalog number 12183018A, Thermo Fisher Scientific). To remove contaminating genomic DNA, DNase treatment (catalog number 12185-010, Thermo Fisher Scientific) was applied on the column. Complementary DNA (cDNA) was generated from an equal amount of total RNA per sample using random hexamers (Applied Biosystems™ High Capacity cDNA Reverse Transcription Kit, catalog number 4374966, Thermo Fisher Scientific). Quantitative PCR (qPCR) was performed as previously described.[35] The relative expression levels of specific genes were analyzed in duplicate and determined using the dCt method with Actin Beta (ACTB) as the endogenous control. The following PrimePCR™ gene-specific primers were purchased from Bio-Rad (Hercules, CA, USA) and the assays performed using the manufacturer's recommend cycling parameters: ACTB (qHsaCED0036269), OSBP (qHsaCID0017727), ORP4 (qHsaCID0012268), ACE2 (qHsaCID0009100), CCL2 (qHsaCID0011608), CCL5 (qHsaCID0011644), CCL20 (qHsaCID0011773), IL1B (qHsaCED0002472), IL6 (qHsaCID0020314), IL8 (qHsaCED0046633), TNF-α (qHsaCED0037461), INFB1 (qHsaCED0019234), IFNL1 (qHsaCED0003353), IFNL2 (qHsaCED0057428), IFNL3 (qHsaCED0038284), CXCL10 (qHsaCED0046619), ISG15 (qHsaCED0001967) and MX1 (qHsaCED0045780). Expression of the SARS-CoV-2 nucleocapsid gene was quantified using the Centers for Disease Control (CDC) designed primers

nCOV_N1 Forward Primer (catalog number 10006821) and nCOV_N1 Reverse Primer (catalog number 10006822) purchased from IDT (San Diego, CA, USA) as described previously.[35] For each time point and condition the gene expression levels were assessed in n=3 replicates with the means used for statistics.

Western blot analysis

Cells were harvested for western blot analysis via direct lysis in the well (following removal of the culture media), with protein lysates run on NuPAGE 4-12% Bis-Tris gradient gels (catalog number NP0336BOX, Thermo Fisher Scientific) using NuPAGE™ MES SDS running buffer (catalog number NP0002, Thermo Fisher Scientific) as previously described.[35] The following primary antibodies were used: OSBP (1:1000 dilution, catalog number sc-365771, Santa Cruz Biotechnology Inc, Dallas, TX, USA), GAPDH (1:5000 dilution, catalog number 2118S, Cell Signaling Technologies, Danvers, MA, USA) and ACE2 (1:1000 dilution, catalog number NBP2-67692, Novus Biologicals, Centennial, CO, USA). The abundance of OSBP (relative to GAPDH levels) was quantified using the ImageJ software (version 1.8.0_112, NIH).

Immunofluorescence staining of SARS-CoV-2 nucleocapsid

BCi-Control and BCi-ACE2 cells (5×10^4) were seeded into chamber slides (catalog number 354114, Corning®) in 1mL of BLEAM. The next day, the cells were infected with SARS-CoV-2 in 0.5mL of BLEAM at a MOI of 0.1 for 2 hours at 37°C. Following infection, the virus inoculum was removed, and the cells were washed three times with 1mL of phosphate buffered saline (PBS) (catalog number 10010023, Thermo Fisher Scientific) then incubated in 1mL of BLEAM. At 48 hours post-infection, the media was removed, and the cells fixed with 10% neutral buffered formalin (catalog number 51201, Expredia™, Kalamazoo, MI, USA) for 20 minutes at room temperature (RT). Following fixation, the cells were permeabilized with 0.1% Triton-X 100

(catalog number 194854, MP Biomedicals, Irvine, CA, USA) for 10 minutes at RT, followed by blocking with 10% goat serum (catalog number 0929391-CF, MP Biomedicals) for 30 minutes at RT. Once blocked, the cells were incubated with primary antibody against SARS-CoV-2 nucleocapsid (10 µg/mL, catalog number MA1-7403, Thermo Fisher Scientific) for 2 hours at RT and then washed three times with PBS, followed by incubation with fluorescently labelled secondary antibody (2 µg/mL, catalog number A11029, Goat anti-mouse Alexa Fluor 488, Thermo Fisher Scientific) for 1 hour at RT. The cell nuclei were counterstained with DAPI (1 µg/mL, catalog number 62248, Thermo Fisher Scientific). Images were taken using an Olympus BX43 upright fluorescent microscope (Olympus Corporation, Tokyo, Japan)

Analysis of SARS-CoV-2 replication kinetics in BCi-ACE2 cells

BCi-ACE2 cells (1×10^5) were seeded into each well of a 12-well plate (catalog number 3513, Corning®) in 1mL of BLEAM. The next day, the cells were either uninfected (mock) or infected with SARS-CoV-2 in 0.5mL of BLEAM at a MOI of 0.1 for 2 hours at 37°C. Following infection, the virus inoculum was removed, and the cells were washed three times with 1mL of PBS then incubated in 1mL of BLEAM. At each time point post-infection (24–72 hour), mock- or SARS-CoV-2-infected cells were collected for RNA extraction and the media for quantification of virus production by $TCID_{50}$ assay using Vero E6-TMPRSS2-T2A-ACE2 cells (catalog number NR-54970, BEI Resources) as previously described.[35] For each independent experiment, experimental conditions and time points were assessed in n=3 replicates with the means used for statistics.

Testing the antiviral effects of OSW-1 treatment against SARS-CoV-2

For experiments investigating the antiviral effects of OSW-1 treatment on SARS-CoV-2 replication, BCi-ACE2 cells (1×10^5) were seeded in an identical manner described above and the next day treated for 24 hours with either DMSO (vehicle control) or 0.1 nM OSW-1. Following

treatment, the cells were washed three times with 1mL of PBS to remove the DMSO/OSW-1, then infected with SARS-CoV-2 at a MOI of 0.1 and harvested at the appropriate time point post-infection (24–72 hour) for quantification of virus replication by qPCR analysis of nucleocapsid expression and production of infectious virus as described above. For each independent experiment, experimental conditions and time points were assessed in n=3 replicates with the means used for statistics.

Stimulation of cells with Poly IC

BCi-NS1.1 cells (1 x 10^5) were seeded into each well of a 12-well plate (catalog number 3513, Corning®) in 1mL of BLEAM. The next day the media was replaced with 1 mL of fresh BLEAM (untreated) or BLEAM supplemented with differing concentrations (0.1, 1 or 10 µg/mL) of Poly IC (catalog number tlrl-pic, InvivoGen, San Diego, CA, USA), and harvested for analysis at 6 and 24 hours post-treatment. For experiments investigating the effect of OSW-1 treatment on the cellular response to Poly IC stimulation, BCi-NS1.1 cells (5 x 10^4) were seeded in an identical manner and the next day treated for 24 hours with either DMSO (vehicle control) or 0.1 nM OSW-1. Following treatment, the cells were washed three times with 1mL of PBS to remove the DMSO/OSW-1, then either untreated or treated with 10µg/mL of Poly IC and harvested for analysis at 6 and 24 hours post-treatment. For each independent experiment, experimental conditions and time points were assessed in n=3 replicates with the means used for statistics.
